# Supplementary material for: Insulin-like Growth Factor 1 Promotes Cell Proliferation by Downregulation of G-Protein-Coupled Receptor 17 Expression via PI3K/Akt/FoxO1 Signaling in SK-N-SH Cells
Source: Int J Mol Sci. 2022 Jan 28;23(3):1513. doi: 10.3390/ijms23031513 (PMC8835821; doi:10.3390/ijms23031513)
Supplement: Supplementary file 1 [file ijms-23-01513-s001.zip › ijms-1514223-supplementary.pdf]

International Journal of
Molecular Sciences

MDPI

Supplemental Materials

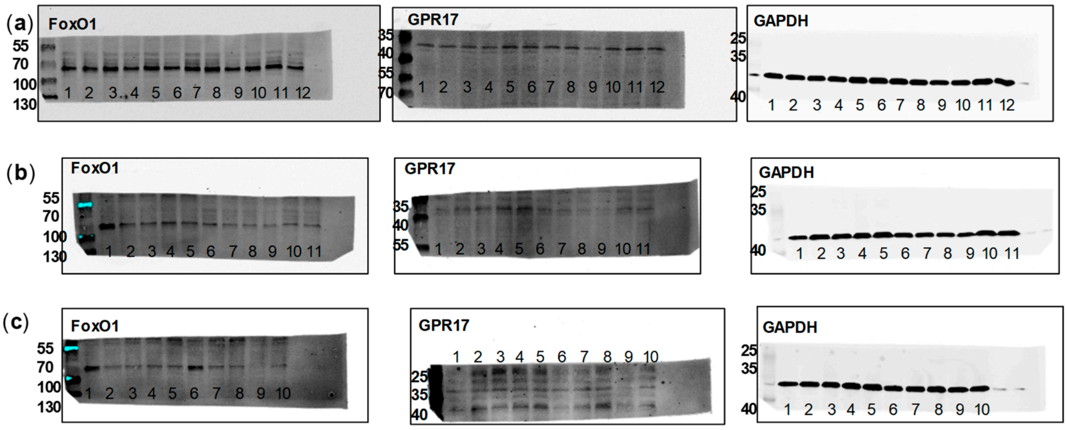

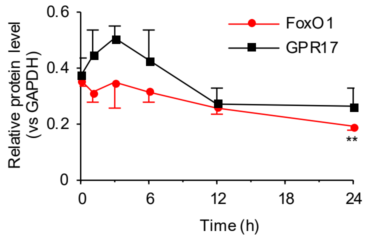

(**d**)

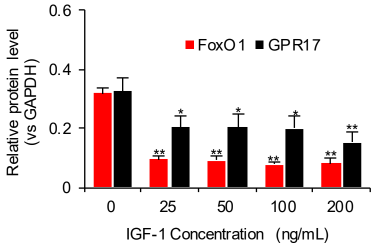

(**e**)

Figure S1. Whole Western blotting results in Figure 1. Each result in Figure 1 was collected from three separate experiments. Western blotting analyses of FoxO1 and GPR17 expression induced by 100 ng/mL IGF-1 within 24 h were presented from channels 1–12 in (**a**) and channels 6–11 in (**b**). Western blotting analyses of FoxO1 and GPR17 protein expression by different IGF-1 concentrations (0, 25, 50, 100, and 200 ng/mL) at 24 h were presented from channels 1–5 in (**b**) and channels 1–10 in (**c**). WB results in Figure 1b were selected from (**a**). The FoxO1 results in Figure 1b were selected from channels 7–12 of (**a**) FoxO1; the GAPHD results in Figure 1b were selected from channels 7–12 of (**a**) GAPHD. WB results in Figure 1e were selected from (**b**). The FoxO1 results in Figure 1e were selected from channels 1–5 of (**b**) FoxO1; the GAPHD results in Figure 1e were selected from channels 1–5 of (**b**) GAPHD. (**d**) Quantification of FoxO1 and GPR17 protein levels induced by 100 ng/mL of IGF-1 within 24 h; (**e**) quantification of FoxO1 and GPR17 protein expression induced by different concentrations of IGF-1 (0, 25, 50, 100, and 200 ng/mL) at 24 h. * $p < 0.05$ vs. 0 h or 0 ng/mL in the IGF-1 group; ** $p < 0.01$ vs. 0 h or 0 ng/mL in the IGF-1 group.

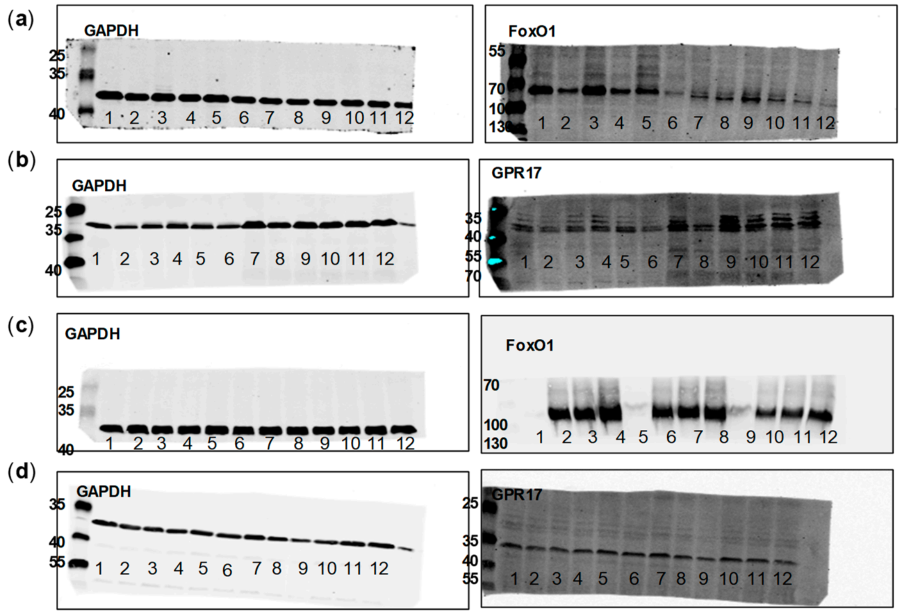

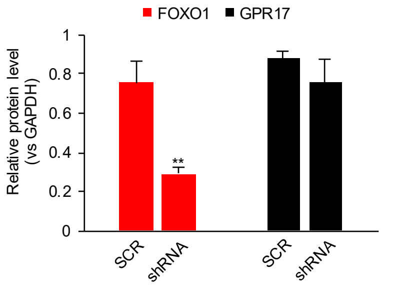

(e)

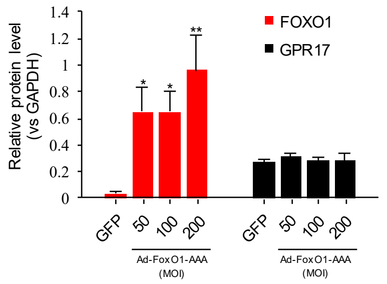

(f)

Figure S2. Whole Western blotting results in Figure 3. Each result in Figure 3 was collected from three separate experiments. Western blotting analyses of FoxO1 and GPR17 expression after FoxO1 silencing were presented from channels 1–6 in (**a**) and channels 1–6 in (**b**). Western blotting analyses of FoxO1 and GPR17 protein expression after FoxO1 overexpression were presented from channels 1–12 in (**c**) and channels 1–12 in (**d**). WB results in Figure 3b were selected from (**a**) and (**b**). The FoxO1 results in Figure 3b were selected from channels 1 and 2 of (**a**) FoxO1; the GAPHD results in Figure 3b associated with FoxO1 were selected from channels 1 and 2 of (**a**) GAPHD; WB results in Figure 3e were selected from (**c**) and (**d**). The FoxO1 results in Figure 3e were selected from channels 9–12 of (**c**) FoxO1; the GAPHD results associated with FoxO1 in Figure 3e were selected from channels 9–12 of (**c**) GAPHD. (**e**) Quantification of FoxO1 and GPR17 protein levels after FoxO1 silencing; (**f**) quantification of FoxO1 and GPR17 protein levels after FoxO1 overexpression. * $p < 0.05$ vs. control; ** $p < 0.01$ vs. control.

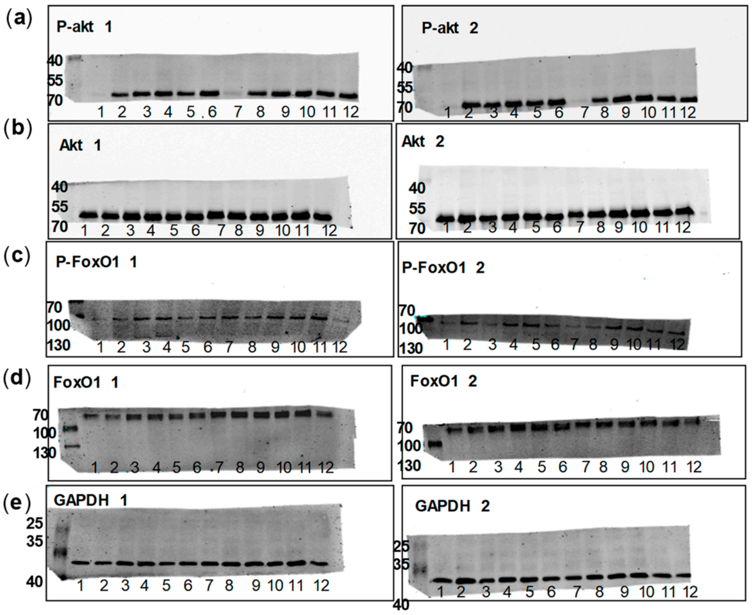

Figure S3. Whole Western blotting results in Figure 4a. Each result in Figure 4a was collected from three separate experiments. Western blotting analyses of P-Akt, Akt, pFoxO1, and FoxO1 protein expression with 100 ng/mL IGF-1 within 60 min were presented from channels 1–12 of the left panel and from channels 1–6 of the right panel. The P-Akt results in Figure 4a were selected from channels 1–6 in the left panel of (**a**) P-Akt; the Akt results in Figure 4a were selected from channels 1–6 in the left panel of (**b**) Akt; the pFoxO1 results in Figure 4a were selected from channels 1–6 in the left panel of (**c**) pFoxO1; the FoxO1 results in Figure 4a were selected from channels 1–6 in the left panel of (**d**) FoxO1; the GAPDH results in Figure 4a were selected from channels 1–6 in the left panel of (**e**) GAPDH.

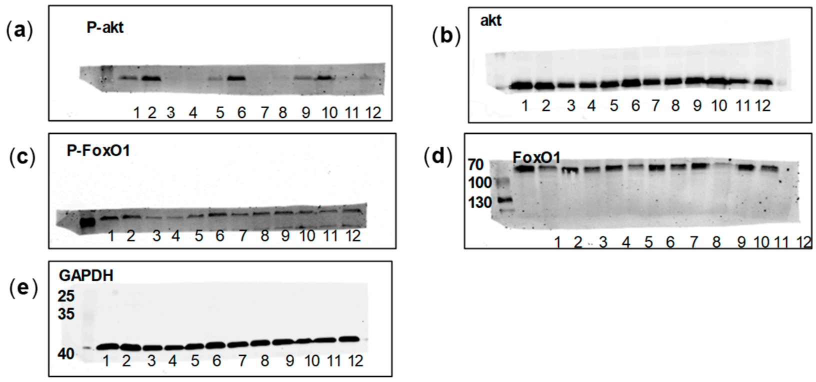

Figure S4. Whole Western blotting results in Figure 4e. Western blotting analyses of P-Akt, Akt, pFoxO1, and FoxO1 expression with or without IGF-1 and/or PI3K inhibitor LY294002 were presented in Figure 4e. The P-Akt results in Figure 4e were selected from channels 5–8 of (**a**); the Akt results in Figure 4e were selected from channels 5–8 of (**b**); the pFoxO1 results in Figure 4e were selected from channels 5–8 of (**c**); the FoxO1 results in Figure 4e were selected from channels 5–8 of (**d**); the GAPDH results in Figure 4e were selected from channels 5–8 of (**e**).

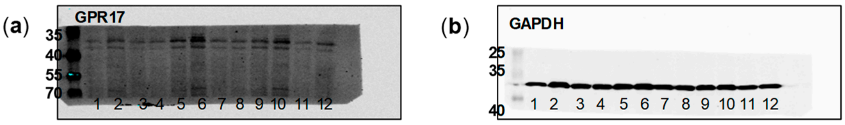

(a)

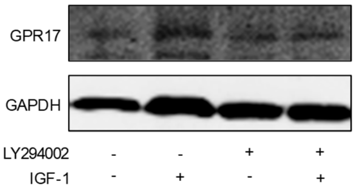

(c)

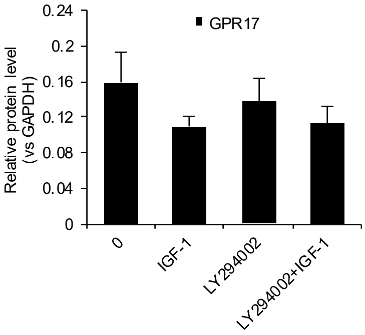

(d)

Figure S5. Whole Western blotting results in Figure 4h. Western blotting analyses of GPR17 expression with or without IGF-1 and/or PI3K inhibitor LY294002 were presented. The GPR17 results in Figure 4h were selected from channels 1–4 of (**a**); the GAPDH results in Figure 4h were selected from channels 1–4 of (**b**). (**c**) Western blot analysis of GPR17 expression in cells with or without IGF-1 and/or PI3K inhibitor (LY294002) treatment; (**d**) quantification of GPR17 levels in cells with or without IGF-1 and PI3K inhibitor (LY294002) treatment.
